# Rigid DNA Frameworks Anchored Transistor Enabled Ultrasensitive Detection of Aβ-42 in Serum

**DOI:** 10.3390/s25113260

**Published:** 2025-05-22

**Authors:** Yungen Wu, Ruitao Lu, Pei-Gen Ren, Zhongjian Xie

**Affiliations:** 1 Institute of Biomedical and Health Engineering, Shenzhen Institute of Advanced Technology, Chinese Academy of Sciences, Shenzhen 518055, China; wuyungen521@163.com; 2 Shenzhen International Institute for Biomedical Research, Shenzhen 518116, China; lucaslu@siitm.org.cn; 3 Shenzhen Children’s Hospital, Shantou University Medical College, Shenzhen 518038, China

**Keywords:** rigid DNA frameworks, tetrahedral DNA nanostructure, field-effect transistor, Aβ-42 detection

## Abstract

It is significant to search for ultrasensitive and accurate testing technology for point-of-care monitoring of common diseases at home; for example, monitoring the Aβ-42 level at any time is crucial for patients suffering from Alzheimer’s disease. However, accurately monitoring the Aβ-42 level in serum is often thwarted by the challenges in sensitivity and specificity due to the multiplicated contaminations and intricated biofluid environments. Here, we develop a graphene field-effect transistor (G-FET) sensor modified with a type of rigid DNA framework aptamer—tetrahedral DNA nanostructure (TDN) for Aβ-42 detection in serum. The Aβ-42 specific aptamer combined with the rigid tetrahedral nanostructure achieves higher binding affinity and better specificity and anti-fouling ability. The detectable concentration reaches 5 × 10^−18^ mol L^−1^ in serum, lower than most other assay approaches. Moreover, the sensor rapidly detects the Aβ-42 level in 6 supernatant samples from mice blood within 5 min and achieves high accuracy. This sensitive and specific method enabled by the DNA tetrahedron G-FET sensor has great potential in the monitoring of Alzheimer’s disease and other diseases.

## 1. Introduction

The severe problem of population aging sharply raises the incidence of Alzheimer’s disease (AD), which seriously threatens the healthy life of elderly people and greatly increases the burden on families worldwide [1,2,3]. AD, as a systemic degenerative disease with insidious onset, is characterized by progressive cognitive decline that can lead to forms of dementia, with a predominance of more than 60% [4,5,6]. The most used diagnostic methods for AD in clinical settings include mental state examination, detection of biomarkers in cerebrospinal fluid (CSF) or plasma and positron emission tomography (PET) imaging or computed tomography (CT) [7,8,9]. However, these technologies are not only time-consuming but also require skilled technicians and specific equipment. Therefore, most study of early identification for AD patients focuses on the precise diagnosis of related biomarkers [10,11,12]. Due to the easy collection, minimal invasiveness and low cost, diagnosis of AD blood biomarkers is preferred and most effective. Aβ-42 is the core biomarker of AD, with strong association with pathological progression, and its levels can be used to differentiate between AD individuals and healthy controls in early clinical stages [13,14,15,16,17]. However, the serum Aβ-42 concentration of early AD patients is relatively low, and traditional methods for Aβ-42 detection in clinics such as ELISA, mass spectrometry and immunofluorescence do not reach the high requirements of sensitivity, specificity and accuracy [18,19,20]. Therefore, developing accurate and convenient diagnostic techniques for AD blood markers that can precisely identify and then develop treatment plans in a timely manner has significant research value in AD study.

Liquid gate field-effect transistor biosensor, as a promising technology for tracing analytes due to its advantages of label-free, easy operation and low reagent consumption, has been widely used for the detection of biomolecules such as proteins, viruses, nucleic acids, cells, etc. Its sensing mechanism works by utilizing the high recognition ability of biomolecules (such as enzymes, antibodies, DNA, cells, etc.) which modifies on the sensing surface, then specific binding target substances (such biomarkers, molecular, ions) combined with rapid signal transduction technology to achieve real-time, sensitive detection [21,22,23]. Graphene is a type of single atomic layer sensing material with the features of high carrier mobility, making it ultra-susceptive to surrounding electrostatic variation, and can output a high sensitivity electric signal. Its advantages of good biocompatibility and easy surface modifiability also make graphene widely applied in bio-detection [24,25,26]. Therefore, the graphene field-effect transistor (G-FET) biosensor has attracted increasing research attention. Compared with ELISA and immunofluorescence methods, the G-FET biosensor has high specificity, ultra-sensitivity and easy portability and integration, which makes it widely applied in medical health, food safety, environmental monitoring and agriculture assay, with significant application prospects in medical diagnosis. However, its application in biomarker detection in complicated biofluid samples is still limited by its unsatisfactory sensitivity and insufficient anti-fouling ability [27,28].

Recent advancements in structural DNA nanotechnology provide an effective way to generate various nanoscale DNA structures with different features and specific functions [29,30,31]. Just like DNA frameworks, especially tetrahedral DNA nanostructure (TDN), the rigid and three-dimensional tetrahedral frameworks result in improved orderliness of probe arrangement and enhanced DNA hybridization efficiency on a biosensing interface by controlling the dimensions of rigid structures [32,33,34]. In DNA aptamers, a sequence of oligonucleotide (DNA or RNA) fragments is obtained from nucleic acid molecular libraries using in vitro screening techniques, Systematic Evolution of Ligands by Exponential Enrichment (SELEX), that have high specificity, affinity and chemical stability [35,36]. As the probe molecule, it can be easy prepared, modified and labeled through chemical synthesis, which exhibits great potential in biofluid detection combined with the structural DNA nanotechnology [37,38,39,40]. Here, we develop a G-FET sensor modified with a tetrahedral DNA framework for Aβ-42 detection in serum. The introduction of a TDN-based aptamer improves the binding affinity and makes G-FET sensors achieve a limit of detection (LoD) down to 5 × 10^−18^ mol L^−1^ for Aβ-42 detection in serum. The sensor exhibits high accuracy within less than 5 min detection time when testing mouse supernatant samples that suffered from Alzheimer’s disease. Owing to its ultra-sensitivity and high accuracy features, the sensor not only provides a convenient and precise technology for monitoring the Aβ-42 levels of Alzheimer’s disease patients but also shows great value in point-of-care diagnosis of other diseases.

## 2. Methods

### 2.1. Graphene Growth

The single-layer graphene sheets were grown via the chemical vapor deposition (CVD) method as the reported literature listed [41]. Firstly, the 25 μm thick and suitable size Cu foils were placed into the tube furnace (GSL 1200X, Hefei Kejing Materials Technology Co., Ltd., Hefei, China) and heated to 1000 °C with 150 sccm flow of Ar (99.0%) for 30 min. Then, the Cu foils were annealed at 1000 °C with a mixture flow of 10 sccm H_2_ (99.999%) and 150 sccm flow of Ar for another 60 min. Then, at 1000 °C, with the atmosphere of 150 sccm Ar, 7 sccm H_2_ and 15 sccm CH_4_ (99.999%) (as the carbon source), they were grown for 15 min. Finally, the CH_4_ flow was cut off, and the Cu foils were immediately cooled down to room temperature; then, they were taken out and placed in a dry environment for later use. At last, the graphene film was transferred to a SiO_2_/Si substrate via electrochemical bubbling method when it was needed.

### 2.2. G-FET Device Fabrication

The G-FET electrode was fabricated via a thermally assisted bilayer lift-off process based on semiconductor IC processing technology. Firstly, spin-coat two layers of resist (sacrificial layer LOR 3A and photoresist S1813) on the SiO_2_/Si wafer sequentially in order to allow for photoresist reflow, then heat-treat on a hot plate at 170 °C for 5 min and 115 °C for 1 min, respectively. Then, using ultraviolet lithography (Microwriter ML3, Durham Magneto Optics Ltd., Cambridge, UK), photoetch the electrode graphic pattern as designed before. Then, we deposited a 5/45 nm Cr/Au on devices via the thermal evaporator (Angstrom Engineering); the photoresist of the place where not photoetched was wiped off by the remover PG stripper solution, and the existing of sacrificial layer LOR 3A layer can tremendously improve the efficiency of lift-off process. Then, the graphene film was transferred onto the device by using a poly(methyl methacrylate) (PMMA) as the carrier layer, and the PMMA was subsequently removed by acetone. Finally, the graphene sensing region (W × L: 30 × 150 μm^2^) was patterned via standard photolithography and etching techniques where graphene on the redundant area was cleared through the oxygen plasma, then washed by the acetone, developing solution and DI water.

### 2.3. TDN Synthesis

The 17bp-5T TDN was synthesized by four single-stranded DNA (ssDNA) through the self-assembly method, and it included three parts: an aptamer sequence which can identify the target molecule, a 5T base as the spacer as the pendant connector and a tetrahedral structure with six double-stranded DNA (ds-DNA) edges. Each vertex of the tetrahedron was modified with an amino group, which makes the TDN link to the graphene surface through chemical bond. All the purified oligonucleotide sequences were purchased from Sangon Biotechnology Inc. (Shanghai, China). Firstly, the DNA power was centrifuged in the tubes at 10,000 rpm at 4 °C for 3 min, then dissolved by suitable volume ultrapure water (18.2 MΩ, Milli-Q system). Then, the concentration of ssDNA stock solutions was tested on the basis of absorbance at 260 nm using UV–visible spectroscopy (Lambda750, Perkin-Elmer, Waltham, MA, USA), then calibrated through the Beer–Lambert law: *A* = *εbc* [42], which makes all the molar quantities at a final concentration (1 μM). Finally, four single-strand DNA for assemblage of the TDN were mixed in 1 × TM buffer at 95 °C for 10 min, cooled to 4 °C immediately using a thermal cycler (SimpliAmp, Thermo Fisher Scientific, Waltham, MA, USA) and stored in 1 × TM buffer at 4 °C for later use.

### 2.4. TDN Functionalization

The TDN probes were immobilized onto the sensing surface by using a chemical linker: 1-pyrenebutanoic acid succinimidyl ester (PASE, Sigma Aldrich, Buchs, Switzerland). The detailed immobilization process follows: firstly, the devices were immersed into acetone solution which contained 5 mM PASE for 12 h at room temperature; then, the devices were washed by acetone, ethanol and DI water three times in turn and dried with nitrogen in the air ambient. Then, a clean and suitable-sized polydimethylsiloxane (PDMS) well was placed onto the sensing area as a container that can hold the target solution; then, the TDN probes were immobilized on the graphene sensing surface, and 50 μL 1 × TM buffer with 200 nM TDN probes was added in the PDMS well and incubated for 12 h at room temperature; after that, the well was rinsed with 1 × TM buffer three times, then added into 80 μL blank buffer and sealed by a piece of glass or wafer and stored at 4 °C in dark for later use.

### 2.5. TDN Characterization

Raman spectrometer (LabRam HR Evolution, Horiba Jobin Yvon, Edison, NJ, USA, 532 nm Ar ion laser) was employed to characterize the graphene before and after PASE modification. The XPS measurements were conducted with a Thermo Fisher Scientific, Waltham, MA, USA, K-Alpha instrument with an Al X-ray source (hν = 1486.6 eV). Then, the sensing surface morphology of the G-FET device after immobilized TDN probes were tested in fluids (1 × TM) by AFM (Fastscan, Bruke, Billerica, MA, USA) operated in ScanAsyst mode using an ultra-sharp tip (Fluid+, Bruke, Billerica, MA, USA) with a 2~3 nm radius. All measured devices were rinsed by ultrapure water and dried by nitrogen before testing. As for the TDN, we used 10% PAGE electrophoresis in 1× TAE buffer (12.5 mM Mg^2+^, pH ~ 7.0) at 100 V for 3 h, then stained in Sybr safe (Thermo Fisher, Waltham, MA, USA) for 30 min, followed by imaging under UV exposure (Gel-doc XR+, Bio-rad, Hercules, CA, USA). As for the confocal fluorescence microscopy measurement, the Cy3-conjugated oligonucleotide strand (A17-5T-Cy3) was introduced to synthesize a fluorescence Cy3-17bp-5T TDN; the control experiment used no fluorescence TDN probes. Then, the devices which immobilized with Cy3-17bp-5T or no fluorescence TDN were put on the confocal microscopy to observe the fluorescence intensity, and the fluorescence was imaged by the confocal fluorescence microscope (C^2+^, Nikon) in 1 × TM buffer.

### 2.6. Preparation of the Test Samples

The pure Aβ-42 powder (1 g) was purchased from Sigma-Aldrich company (St. Louis, MO, USA). Firstly, the initial liquor with a concentration of 1 mM was prepared. Then, the 1 mM initial liquor was serially diluted in PBS or serum to concentrations of 5 × 10^−10^, 5 × 10^−11^, 5 × 10^−12^, 5 × 10^−13^, 5 × 10^−14^, 5 × 10^−15^, 5 × 10^−16^, 5 × 10^−17^, 5 × 10^−18^ and 5 × 10^−19^ mol L^−1^ to store for later use. All the diluted samples were centrifuged at 3000 rpm for 1 min to make sure the target was fully dissolved before testing, which can facilitate specific capture by the TDN G-FET sensor. Then, we removed the blank medium in the PDMS well, adding the different concentration target solution immediately. Then, we incubated for 15 min and recorded the changes in electrical signals. The supernatant serum samples were collected from the feeding mice whole blood by centrifuging at 3000 rpm for 5 min, then detected by the TDN G-FET sensor that we developed.

### 2.7. Device Measurement

The source-drain electrodes were wire-bonded by Al wires. When we measured the transfer curve of the G-FET devices, 100 μL buffer solution was added into the PDMS well, and an Ag/AgCl reference electrode was inserted into the solution as the liquid gate electrode. The electrical characterization was carried out at room temperature using a semiconductor analyzer (Keysight, Santa Rosa, CA, USA, B1500A). Each sample of different concentration was measured at an interval of 15 min which guaranteed that the target insulin was bonded to the TDN probes. To add testing solutions, 50% volume solution was taken out from the PDMS well and replaced by the same volume of pre-mixed samples. Because the *V*_ds_ was stabilized (50 mV), *I*_ds_ versus *V*_lg_ curves were obtained by sweeping *V*_lg_ in a certain range, and the Dirac point was obtained at a *V*_lg_ where Ids reaches its minimum. Once the probes were bonded to the target insulin, a doping effect was exerted to the graphene surface, inducing a Dirac point shift compared to the previous result through measuring the transfer curves. As for the real-time *I*_ds_ − t measurement, the target insulin solution concentration change can induce an *I*_ds_ decrease or increase compared to the previous concentration.

### 2.8. Animal Model and Preparation of Supernatant Samples

All animal experimental protocols were carried out according to the guidelines approved by the Animal Care and Use Committee of the Shenzhen Institute of Advanced Technology Chinese Academy of Sciences. First, mice (4- to 5-month-old male or female 5 × FAD mice) were maintained in a sterile environment with light, humidity and temperature control. After feeding for several months, we picked six mice in good condition. Then, we took blood from their eyes and added an appropriate amount of anticoagulant, followed by stewing for 2–3 h and placing in a high-speed centrifuge at 12,000 rpm/min for centrifugation 5 min. After that, the supernatant serum samples were taken out and stored for later testing.

### 2.9. Limit of Detection (LoD) and Relative Standard Deviation (RSD) Calculation

The LoD calculation follows the following formula:

$$LoD=\frac{3\sigma }{S}$$

where *σ* is the standard deviation of testing noise, and *S* is the sensitivity acquired as follows:

$$S=\frac{I_{ds}^{\prime }-I_{ds0}}{c^{\prime }-0}$$

where *c’* is the tested concentration; *I*’_ds_ is the current response corresponding to *c’*; and *I*_ds0_ is the current response corresponding to negative control.

$$RSD=\frac{SD}{\bar{X}}\times 100\%$$

where *SD* is the standard deviation, and $\bar{X}$ is the average of calculated values.

## 3. Results and Discussion

**Figure 1:**
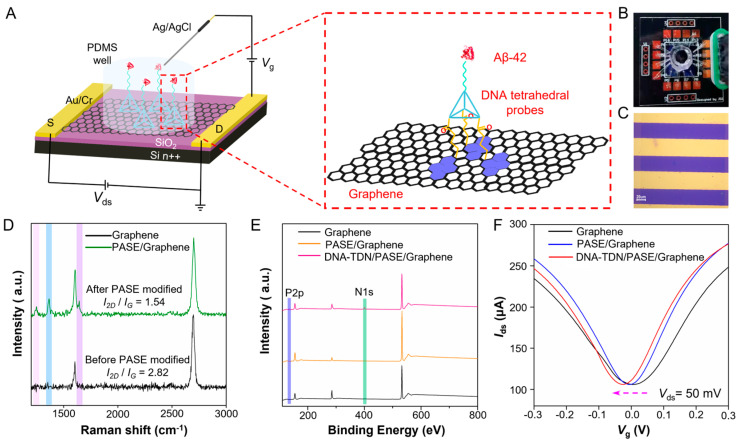
**TDN G-FET sensor for Aβ-42 testing.** (**A**) Workflow and schematic diagram of the TDN G-FET sensor for Aβ-42 testing. Enlarged diagram is the G-FET sensing surface modified with TDN probes. (**B**) Digital photograph of working device. (**C**) Optical microscope image of the G-FET channel. (**D**) Raman spectrum of bare graphene and graphene after PASE modified. (**E**) XPS measurement of bare graphene, graphene after PASE modified and graphene after TDN immobilized. (**F**) *I*_d_-*V*_g_ measurement of bare graphene device, graphene device that after PASE modified, and graphene device after TDN immobilized. (**D**–**F**) Measurements suggest the graphene has monolayer and highly quality characteristics and provide direct or indirect evidence that the PASE and TDN probes are modified on the sensing surface successfully.

The workflow of the TDN G-FET sensor for Aβ-42 detection is illustrated in Figure 1A. It is composed of a liquid-gated G-FET with a polydimethylsiloxane (PDMS) chamber on the graphene channel (Figure 1B) with tetrahedral DNA probes on the sensing interface (enlarged diagram). Each worked sensing area (Figure 1C) is 30 × 120 μm^2^ (*W* × *L*). The designed DNA nanostructure is an Aβ-42 specific aptamer grafted on a tetrahedron with the edge length of 17 base pair (~5.3 nm of theoretical length) and a 5T space (Figure S2A). All the DNA sequences (5′-3′) are listed in Table S1 (Supporting Information). The synthesized DNA tetrahedron nanostructures are analyzed by 10% polyacrylamide gel electrophoresis; most of the lanes only have a major band, and the yield is calculated as ~90% (Figure S2B), which can confirm successful synthesis of the DNA tetrahedron. The 1-pyrenebutanoic acid succinimidyl ester (PASE) is a molecule linker that can help DNA tetrahedral probes immobilizing onto the sensing surface of the G-FET device (Figure S3). Here, the Aβ-42 specific aptamer is targeted to Aβ-42 peptide, as a monomer that can be polymerized to an oligomer, which is a conformation-specific spatial structure produced by enzymatic hydrolysis. The spatial structure of Aβ oligomer is very different from the Aβ monomer and has a strong association with pathological progression. The Raman spectrum (Figure 1D) test shows that the graphene was single-layer with high quality before being modified and remains homogeneous after PASE modification through the π−π interaction. The mapping image of graphene after PASE modification (Figure S1A,B) also reveals that the graphene is homogeneous. The fast scan atomic force microscopy (AFM) test in fluid reveals that TDN probes anchored on graphene surface successfully with an orderly manner (Figure S4A), and the surface roughness observably increases after anchorage. Confocal fluorescence microscopy measurement results (Figure S4B) also indicate that the Cy3-17bp-5T TDN successfully immobilized on the sensing surface. The X-ray photoelectron spectroscopy (XPS) test result shows that the N1s peak and P2p peak (Figure 1E and Figure S5A,B) appeared, which furtherly verified that the PASE and TDN probes were modified on the graphene sensing surface successfully. Furthermore, the transfer curve measurement (Figure 1F) of the device indicated that the *V*_Dirac_ (the liquid-gate voltage *V*_lg_ when the drain-source current *I*_ds_ reaches its minimum) gave rise to a negative offset after PASE modification, due to the n-doping effect to graphene induced by the π−π interaction, and a more negative shift occurred after TDN probe immobilization. All the above characterizations concluded that the high-performance sensing surface of G-FET has been successfully constructed.

**Figure 2:**
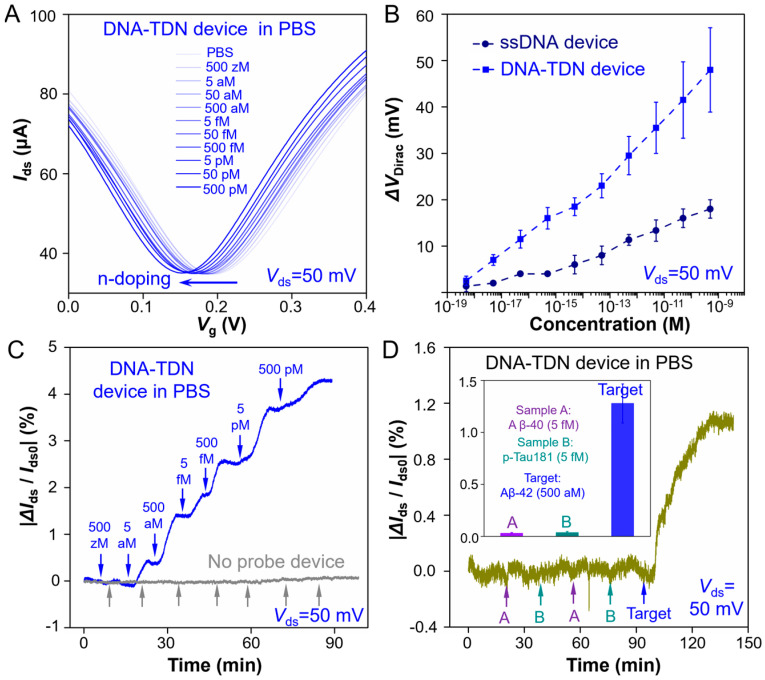
**TDN G-FET sensor for Aβ-42 testing in PBS**. (**A**) Transfer curve measurement of adding different concentration Aβ-42 in PBS buffer (*I*_ds_ − *V*_lg_ response curve). (**B**) ∆*V*_Dirac_ response upon different concentration Aβ-42 in PBS buffer of TDN probes modified (blue line) and ssDNA probes modified (navy blue line) G-FET device. (**C**) Real-time |∆*I*_ds_/*I*_ds0_| response upon different concentration Aβ-42 in PBS buffer (blue line: modified with TDN probes; gray line: without immobilized any probes). (**D**) |∆*I*_ds_/*I*_ds0_| responses of TDN G-FET sensor to Aβ-42 in PBS (5 × 10^−16^ mol L^−1^) and the proteins which are homologous to Aβ-42 (sample A: 5 × 10^−15^ mol L^−1^ Aβ-40, sample B: 5 × 10^−15^ mol L^−1^ p-Tau 181). The inset histogram is the error bars of (**B**,**D**) defined by the standard deviation of the results from at least 3 parallel experiments. All Aβ-42 solutions samples were dissolved in PBS buffer with addition of 12.5 mM Mg^2+^ and pH is 7.0~7.4.

The TDN G-FET sensor for Aβ-42 measuring capabilities is first checked in 1 × PBS buffer. We test the transfer curve (*I*_ds_ − *V*_lg_) of the G-FET device and record the *V*_Dirac_ shift when adding different Aβ-42 solution concentrations to the PDMS chamber. Here, *I*_ds_ means the output real-time drain-source current, *V*_lg_ means the liquid-gate voltage of the G-FET device and *V*_Dirac_ represents the *V*_lg_ when *I*_ds_ reaches its minimum. As shown in Figure 2A, when the Aβ-42 solution concentration increases, more Aβ-42 was binding to the aptamer probes, making the measured *V*_Dirac_ left shift at a variable rate within physiologically relevant Aβ-42 levels. It is because that binding of Aβ-42 to the aptamer could generate n-type doping to graphene and then induce a significant *V*_Dirac_ shift of the G-FET device. The aptamers we choose in this study were targeted to the Aβ-42 specific aptameric receptor. When the target Aβ-42 solution concentration increases from 500 zM (5 × 10^−19^ mol L^−1^) to 500 pM (5 × 10^−10^ mol L^−1^), the *V*_Dirac_ shows a continues negative shift and totally shifts about 50 mV when adding to 500 pM Aβ-42 solutions. The TDN G-FET shows a significant electrical response signal and excellent measuring capabilities to the target Aβ-42. Compared to the electrical response signal of the ssDNA aptamer immobilized G-FET sensor (Figure S6A), the response of the TDN G-FET shows more greater response signal and better linear relationship (Figure 2B).

Then, the real-time electrical responses (*I*_ds_ − *t*) of the TDN G-FET sensor to Aβ-42 solution are tested (Figure 2C) at different concentrations from 500 zM to 500 pM. Remarkable electrical response signals (|∆*I*_ds_/*I*_ds0_|) are detected within 5 min even when target Aβ-42 solution concentration is 5 aM (5 × 10^−18^ mol L^−1^). Here, we define the response values |∆*I*_ds_/*I*_ds0_| = |(*I*_ds_ − *I*_ds0_) / *I*_ds0_|, where *I*_ds_ is the real-time drain-source current, and Ids0 is the initial current. As for the sensor without immobilized probes, no obvious response signals (Figure 2C) are observed even when 500 pM (5 × 10^−10^ mol L^−1^) solution is added (gray line). Then, the |∆*I*_ds_/*I*_ds0_| responses of the ssDNA immobilized G-FET device to Aβ-42 solution are also measured under the same conditions, and the |∆*I*_ds_/*I*_ds0_| response values are recorded in Figure S6B. Compared to the ssDNA aptamer sensors, the |∆*I*_ds_/*I*_ds0_| response of the TDN G-FET (Figure 2D) sensor is larger, and the response time (<5 min) is also much shorter than that of the ssDNA sensor (~15 min). In addition, from the |∆*I*_ds_/*I*_ds0_| response values of the TDN and ssDNA immobilized G-FET sensors, we calculated that the theoretical LoD of the TDN G-FET sensor reaches 5 aM. It benefited from the high binding affinity and favorable anti-fouling capability of the TDN, which enables more efficient binding to target Aβ-42, then presented a larger |∆*I*_ds_/*I*_ds0_| response value and shorter response time in Ids − t measurement, as well as a larger *V*_Dirac_ offset in *I*_ds_ − *V*_lg_ measurement. Moreover, the ssDNA probes immobilized on the graphene sensing surface will largely produce mutual entanglement and adsorption on graphene, which will reduce the number of effective probes and significantly decrease the electrical response signals.

Then, we measured the *I*_ds_ − *t* response values by adding other two non-targeted samples with concentrations of 5 fM (5 × 10^−15^ mol L^−1^) in PBS, including Aβ-40 (sample A) and p-Tau 181 (sample B) proteins. The |∆*I*_ds_/*I*_ds0_| response values of non-targeted samples (inset figure in Figure 2D) are negligible compared to that of the target Aβ-42 sample even when the added concentration is 500 aM (5 × 10^−16^ mol L^−1^). Thus, we conclude that the TDN modified G-FET sensor not only exhibits excellent detection performance of ultra-sensitivity and short response time but also shows high specificity.

It is well known that bio-detection includes a bio-recognition and signal transduction process [33,34,37,38,39,40]. The binding affinity between DNA probe and target is the crucial factor in the bio-recognition process. The signal transduction process could be amplified through the G-FET device and reflected on the electrical measurement, which mainly reveals in the *V*_Dirac_ offset of transfer curve test and the current change of real-time measurement. The binding process can induce charge accumulation on graphene surface, then cause a doping effect to graphene, and the monolayer graphene with high mobility enables efficient and sensitive signal transduction process, which is the fundamental reason for the TDN G-FET sensor achieving such an excellent detection performance.

Here, we calculate the binding affinity by using normalized response of ∆*V*_Dirac_/∆*V*_Dirac,max_; the correlation between ∆*V*_Dirac_/∆*V*_Dirac,max_ and target analyte concentration is described by the Hill–Langmuir model:

$$\frac{∆V_{Dirac}}{∆V_{Dirac,max}}=A\frac{{\left(C_{analyte}/K_{D}\right)}^{n}}{1+{\left(C_{analyte}/K_{D}\right)}^{n}}$$

where ∆*V*_Dirac,max_ is the maximum ∆*V*_Dirac_, denoted as ∆*V*_Dirac,max_ = *V*_Dirac,max_ − *V*_Dirac,0_ (*V*_Dirac,0_ and *V*_Dirac,max_ refer to the offset of adding the zero and maximum concentration target analyte solution, respectively); *A* is the saturation response coefficient of the sensing system; and *n* is the Hill coefficient corresponding to the binding cooperativity. Thus, the pseudo *K*_D_ of the TDN G-FET sensor is 1~3 × 10^−18^ mol L^−1^ and could be calculated from the fitted *I*_ds_ − *V*_lg_ responses curve, which is five orders of magnitude lower than that of the ssDNA aptamer G-FET sensor. The results reveal that the TDN G-FET sensor has a larger offset of ∆*V*_Dirac_ than that of ssDNA sensors. The TDN enables a higher binding affinity and shorter bio-recognition time compared to the ssDNA probes. In addition, unlike ssDNA probes, the existence of a tetrahedral structure can effectively prevent the probe DNA intertwining with each other and avoid the non-specific bio-molecule adsorption on the graphene surface. Thus, the special structure of the TDN combined with efficient signal transduction of the G-FET gives rise to ultra-sensitive detection of analyte.

**Figure 3:**
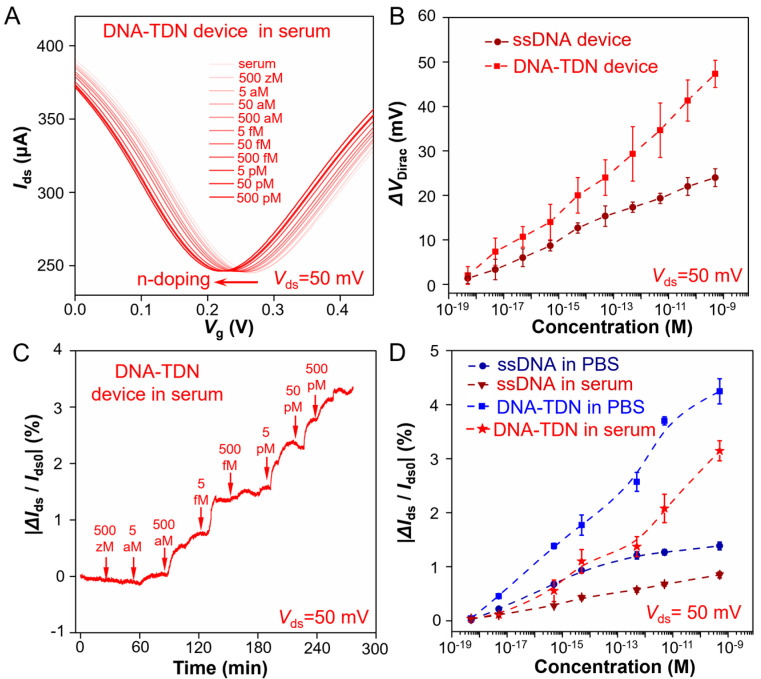
**TDN G-FET sensor for Aβ-42 testing in serum.** (**A**) Transfer curve measurement of adding different concentration Aβ-42 in serum (*I*_ds_ − *V*_g_ response curve). (**B**) ∆*V*_Dirac_ response upon different concentration Aβ-42 in PBS buffer of TDN probes modified G-FET device (red line) and ssDNA probes modified G-FET device (crimson line). (**C**) Real-time |∆*I*_ds_/*I*_ds0_| response upon different concentration Aβ-42 in serum. (**D**) |∆*I*_ds_/*I*_ds0_| responses of TDN probes and ssDNA probes G-FET sensor to Aβ-42 in PBS or in serum. The error bars of (**B**,**D**) are defined by the standard deviation of the results from at least 3 parallel experiments. All Aβ-42 solutions samples were dissolved in PBS buffer with addition of 12.5 mM Mg^2+^ and pH is 7.0~7.4.

The ultraprecise analysis capability of trace analytes in complicated biofluids is of great significance for biological research. Thus, the TDN G-FET sensor for Aβ-42 detection performance in full serum is also evaluated. We first test the transfer curve (*I*_ds_ − *V*_lg_) of the G-FET device and record the *V*_Dirac_ shift when adding different Aβ-42 solution concentrations. Figure 3A shows that with the Aβ-42 solution concentration increased, the *V*_Dirac_ of the TDN G-FET device occurs with a continues left shift, which is larger than that of the ssDNA immobilized device (Figure S7A). The statistical *V*_Dirac_ shifts of all Aβ-42 concentration in Figure 3B are collected by three parallel experiments that were in the same condition. The real-time electrical responses (*I*_ds_ − *t*) of the TDN G-FET sensor to Aβ-42 solutions is measured (Figure 3C) at different concentrations, and the |∆*I*_ds_/*I*_ds0_| responses (*I*_ds_ − *t*) of the ssDNA G-FET sensor to all concentration (Figure S7B) are recorded. From the statistics |∆*I*_ds_/*I*_ds0_| response values of all Aβ-42 concentrations (Figure 3D) through three parallel experiments, it is revealed that the TDN G-FET sensor has ultraprecise and sensitive detection capability for Aβ-42 in full serum. To further confirm the interfering molecules in the serum, such as proteins, lipid or DNA, which cannot disturb the function of this biosensor, we conducted the relevant selectivity experiments that test the transfer curve of this G-FET sensor and observed the shift of the *V*_Dirac_ point when adding serum and random DNA sequences that dissolved in serum. The results are shown in Figure S8. These testing results demonstrate that the serum proteins, lipid or DNA cannot disturb the function of this biosensor, and it validates that the sensor has excellent detection performance and reliability in serum. This is beneficial for at-home monitoring for the Aβ-42 level at any time, which is crucial for patients who suffer from Alzheimer’s disease.

**Figure 4:**
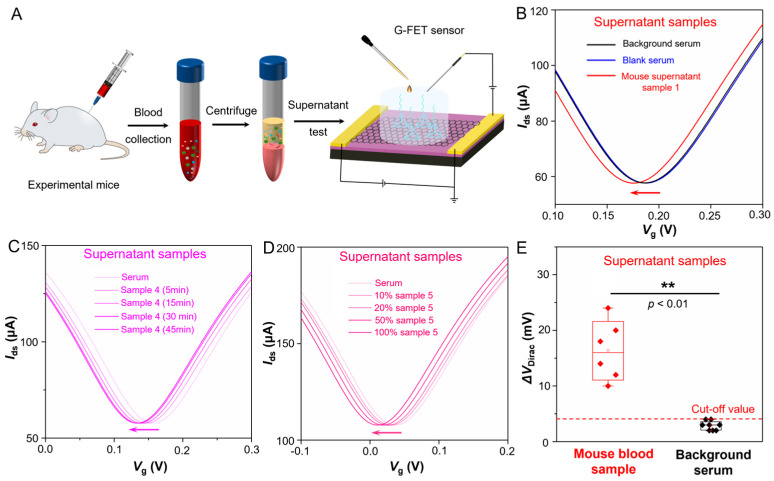
**TDN G-FET sensor for Aβ-42 testing from supernatant samples of experimental mice.** (**A**) Schematic diagram of TDN G-FET sensor for Aβ-42 testing of supernatant samples. (**B**) |∆*V*_Dirac_| response upon addition of blank serum and mouse supernatant samples with Alzheimer’s disease. (**C**) |∆*V*_Dirac_| response upon addition of mouse supernatant samples with Alzheimer’s disease by measured after 5 min, 15 min, 30 min and 45 min. (**D**) |∆*V*_Dirac_|response upon addition of mouse supernatant samples with Alzheimer’s disease by diluting 10 time (10%), 5 time (20%), 2 time (50%) and 1 time (100%). (**E**) |∆*V*_Dirac_| response upon addition of mouse supernatant samples with Alzheimer’s disease (positive sample 1~6) and blank serum (negative sample 1~8).

Furthermore, we tested six supernatant samples (Sample 1~6) collected from the blood serum of laboratory-cultured mice which suffering from Alzheimer’s disease. The schematic diagram of TDN G-FET sensor for Aβ-42 testing of supernatant samples is shown in Figure 4A. The real-time |∆*V*_Dirac_| response of the G-FET sensor upon adding blank serum and supernatant sample 1 is shown in Figure 4B; the |∆*V*_Dirac_| response result reveals that unlike adding the blank serum, once adding supernatant sample 1, the ∆*V*_Dirac_ of this sensor occurs with a large left shift. Due to the TDN probes immobilized on the sensing surface binding to the target Aβ-42 in supernatant, then induces a n-doping to the graphene and makes the ∆*V*_Dirac_ left shifting. Then we test the |∆*V*_Dirac_| response of G-FET sensor upon adding supernatant sample 4 with different reaction time (5 min, 15 min, 30 min and 45 min), the result (Figure 4C) shows that even though with 5 min reaction time, the TDN probes also can exactly and rapidly binding to the target Aβ-42 in the complex serum environment and induce a noticeable shifted. This sensor exhibits a less than 5 min response time in the supernatant samples owing to the TDN probes immobilized onto the sensing surface sequenced and orderly. After that, we test the |∆*V*_Dirac_| response of G-FET sensor when adding supernatant sample 5 with several concentration gradients which diluted in ten times, five times, two times and entire concentration, the result (Figure 4D) shows that even though the supernatant sample that diluted in ten times, the ∆*V*_Dirac_ occurs a remarkable negative shift with 5 min, which reveals this G-FET sensor achieves a sensitive and precise detecting capability to insulin in supernatant serum samples. Then, all six supernatant samples are tested by the G-FET sensors (Figure S9A–E) and the statistical |∆*V*_Dirac_| responses of supernatant samples and blank serum samples are recorded in Figure 4E and Table S2. The above test shows this TDN G-FET sensor can rapidly and sensitively detect the Aβ-42 in the blood serum supernatant, it can accurately distinguish between blank serum samples and blood serum supernatant samples that containing Aβ-42, which exhibits excellent detection performance and great application potential in home monitoring of Alzheimer’s disease.

## 4. Conclusions

In this work, we develop a G-FET sensor modified with TDN probes which realizes rapid and sensitive detection of Aβ-42 in serum, as well as in blood serum supernatant samples. The high binding affinity of aptamer combined with the appropriate size and rigid structure of TDN frameworks constructs a high-performance sensing interface, making the detectable concentration of TDN G-FET for Aβ-42 detection down to 5 × 10^−18^ mol L^−1^ in full serum with less than 5 min. It overcomes the extrinsic detection defects of limited sensitivity and insufficient accuracy which were caused by high amounts of ‘background’ biomolecules when employing it in complex biofluid detection, and it achieves a rapid, precise detection capability. The sensor also can exactly identify the blood serum supernatant sample which contains target Aβ-42 despite its low concentration and the blank serum sample, which reveals great values to solve the problem of point-of-care detection for patients who suffered from Alzheimer’s disease, as well as monitoring the Aβ-42 level at any time at home. However, the sensing technology we developed may have shortcomings such as insufficient stability, lack of clinical sample verification and inability to be truly applied to POC at this stage. More in-depth research needs to be conducted to overcome these shortcomings when integrated with a portable microelectronic system and can make this new technology be developed into a comprehensive platform, which can rapidly and accurately monitor other diseases via replacing the aptamer. We believe that this rapid, easily-operated and accurate detection technology provides a new insight for development of novel diagnostic equipment and will be beneficial for accurate diagnosis of various diseases.

## Data Availability

Data are contained within the article and Supplementary Materials.

## Supplementary Materials

The following supporting information can be downloaded at: https://www.mdpi.com/article/10.3390/s25113260/s1, Figure S1: Raman characterization of graphene and after PASE modified. (A), *I*_2D_/*I*_G_ mapping image of bare graphene. (B), *I*_2D_/*I*_G_ mapping image of graphene after PASE modification. Figure S2: Synthesis and characterization of 17bp-5T TDN probes. (A), Synthetic route of 17bp-5T-IGA3 tetrahedral probes by four DNA chain designed. (B), PAGE (10%) characterization of the DNA tetrahedron and ssDNA. lane 1: 25-500 bp DNA marker; lane 2: ss DNA chain A; lane 3: ssDNA chain A and B; lane 4: ssDNA chain A, B and C; lane 5: ssDNA chain A, B, C and D; lane 6: ssDNA chain D. The results demonstrated that the 17bp-5T-IGA3 tetrahedral probes were synthesized successfully. Figure S3: TDN immobiliziton onto the graphene. PASE (5mM/L) was dissolved in acetone and immobilized onto the graphene surface by π-π stacking for 2 h. Then, TDN probes is covalently bonded to PASE on graphene surface by amidation for 12 hours in 1×TM buffer solution. Figure S4: AFM characterization and confocal fluorescence microscopy measurement of sensing surface morphology. (A), AFM image of the graphene surface that modified with PASE and immobilized with TDN probes. (B), Confocal fluorescence microscopy image of the graphene surface that modified with PASE and immobilized with Cy3-conjugation TDN. Figure S5: XPS measurement. (A) N1s and (B) P2p spectra of bare graphene (black), graphene after modification with PASE (orange) and after immobilization of TDN on PASE (pink). The appearance of N1s peak and P2p indicates successful immobilization of PASE and TDN, respectively. Figure S6: ssDNA probes modified G-FET device detection the insulin in PBS. (A), Transfer curve measurement of adding different concentration insulin in PBS (*I*_ds_−*V*_g_ response curve) by the ssDNA probes modified G-FET device. (B), Real-time |∆*I*_ds_/*I*_ds0_| response upon different insulin in PBS of the ssDNA probes modified G-FET device. Figure S7: ssDNA probes modified G-FET device detection the insulin in serum. (A), Transfer curve measurement of adding different concentration insulin in serum (*I*_ds_−*V*_g_ response curve) by the ssDNA probes modified G-FET device. (B), Real-time |∆*I*_ds_/*I*_ds0_| response upon different insulin in serum of the ssDNA probes modified G-FET devic. Figure S8: Transfer curve measurement of adding serum, random DNA in serum and Aβ-42 in serum (*I*_ds_−*_V_*_g_ response curve). Figure S9: TDN G-FET device detection the insulin in supernatant samples from feeding mice. |∆*V*_Dirac_| response of TDN G-FET sensor when adding different mouse supernatant samples (figure A, B, C, D, E and F are correspondenced Sample 1, 2, 3, 4, 5 and 6, respectively). Table S1. The sequences (5′-3′) of DNA in this work. Table S2. ∆*V*_Dirac_ values measured by the TDN G-FET sensor for insulin test from the supernatant of experimental mice bloods.
