# Supplementary material for: Rigid DNA Frameworks Anchored Transistor Enabled Ultrasensitive Detection of Aβ-42 in Serum
Source: Sensors (Basel). 2025 May 22;25(11):3260. doi: 10.3390/s25113260 (PMC12158189; doi:10.3390/s25113260)
Supplement: Supplementary file 1 [file sensors-25-03260-s001.zip › sensors-3595455-supplementary.pdf]

Supporting Information (SI)

DNA tetrahedron based transistor enabled ultrasensitive detec-tion of Aβ-42 in serum

Yungen Wu [1,2], Ruitao Lu[2], Pei-Gen Ren[1,*], Zhongjian Xie[3,*]

[1] Institute of Biomedical and Health Engineering, Shenzhen Institute of Advanced Technology Chinese Acad-emy of Sciences, Shenzhen, 518055, China;

[2] Shenzhen International Institute for Biomedical Research, Shenzhen 518116, Guangdong, China;

[3] Institute of Pediatrics Shenzhen Children's Hospital, Clinical Medical College of Southern University of Sci-ence and Technology, Shenzhen 518038, Guangdong, P. R. China. xiezj2022@sustech.edu.cn

SUPPORTING FIGURES

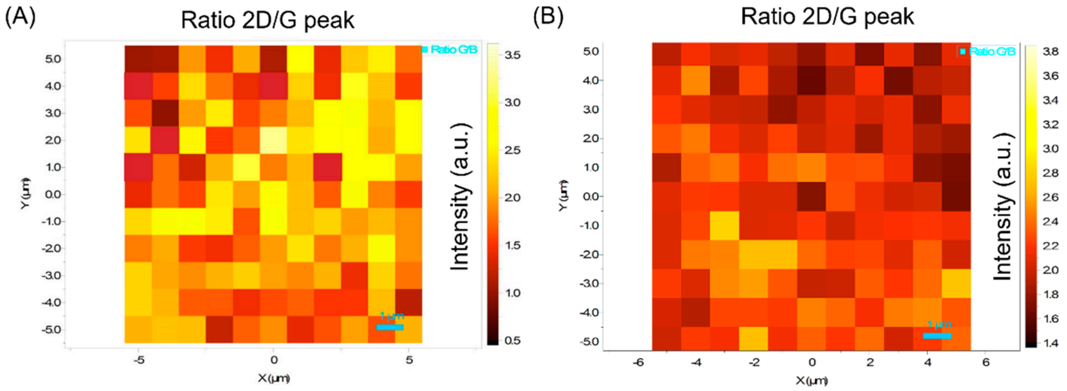

Figure S1: Raman characterization of graphene and after PASE modified. (A), I_{2D}/I_G mapping image of bare graphene. **(B),** I_{2D}/I_G mapping image of graphene after PASE modification.

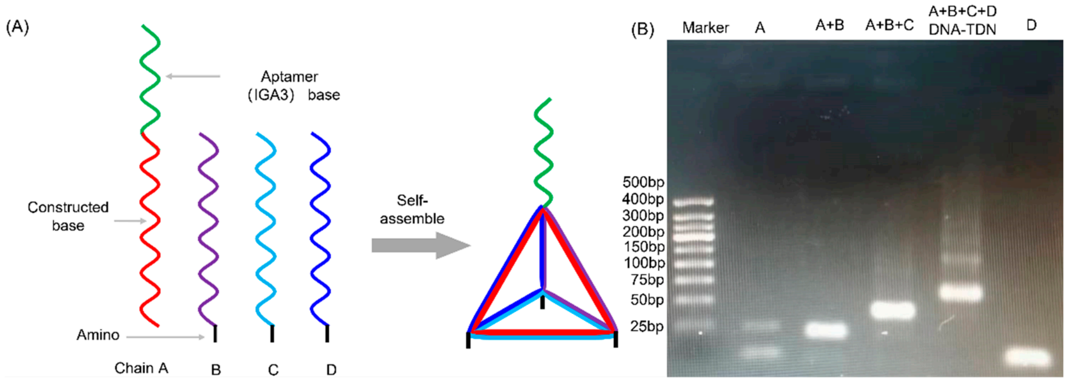

Figure S2: Synthesis and characterization of 17bp-5T TDN probes. (A), Synthetic route of 17bp-5T-IGA3 tetrahedral probes by four DNA chain designed. **(B),** PAGE (10%) characterization of the DNA tetrahedron and ssDNA. lane 1: 25-500 bp DNA marker; lane 2: ss DNA chain A; lane 3: ssDNA chain A and B; lane 4: ssDNA chain A, B and C; lane 5: ssDNA chain A, B, C and D; lane 6: ssDNA chain D. The results demonstrated that the 17bp-5T-IGA3 tetrahedral probes were synthesized successfully.

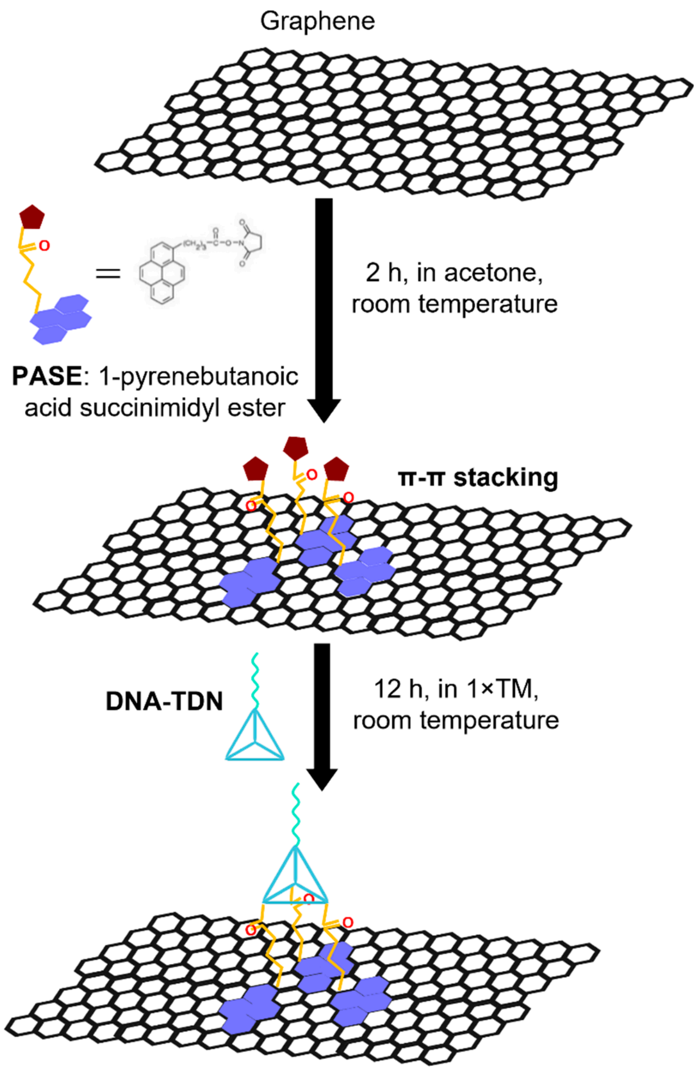

Figure S3: TDN immobiliziton onto the graphene. PASE (5mM/L) was dissolved in acetone and immobilized onto the graphene surface by π-π stacking for 2 h. Then, TDN probes is covalently bonded to PASE on graphene surface by amidation for 12 hours in 1×TM buffer solution.

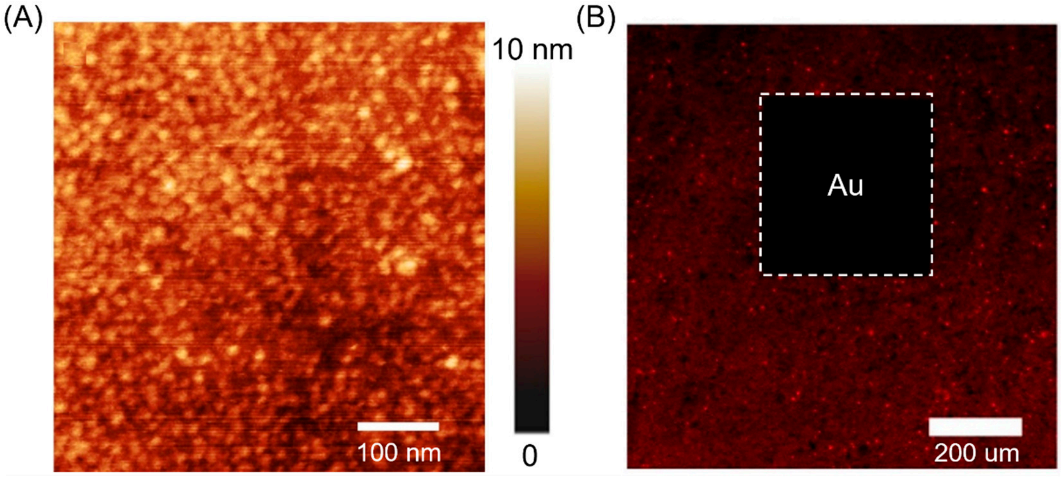

Figure S4: AFM characterization and confocal fluorescence microscopy measurement of sensing surface morphology. (A), AFM image of the graphene surface that modified with PASE and immobilized with TDN probes. **(B),** Confocal fluorescence microscopy image of the graphene surface that modified with PASE and immobilized with Cy3-conjugation TDN.

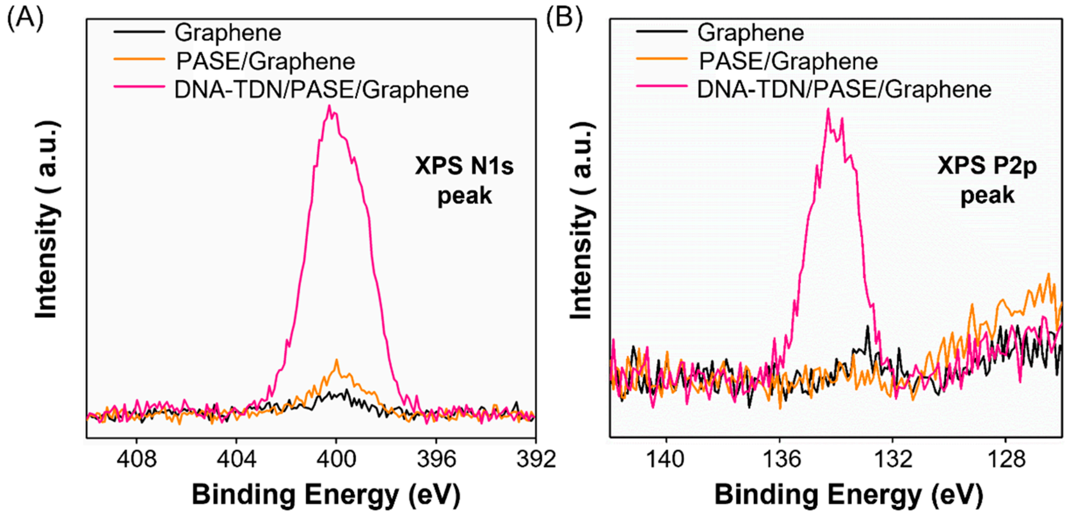

Figure S5: XPS measurement. (**A**) N1s and (**B**) P2p spectra of bare graphene (black), graphene after modification with PASE (orange) and after immobilization of TDN on PASE (pink). The appearance of N1s peak and P2p indicates successful immobilization of PASE and TDN, respectively.

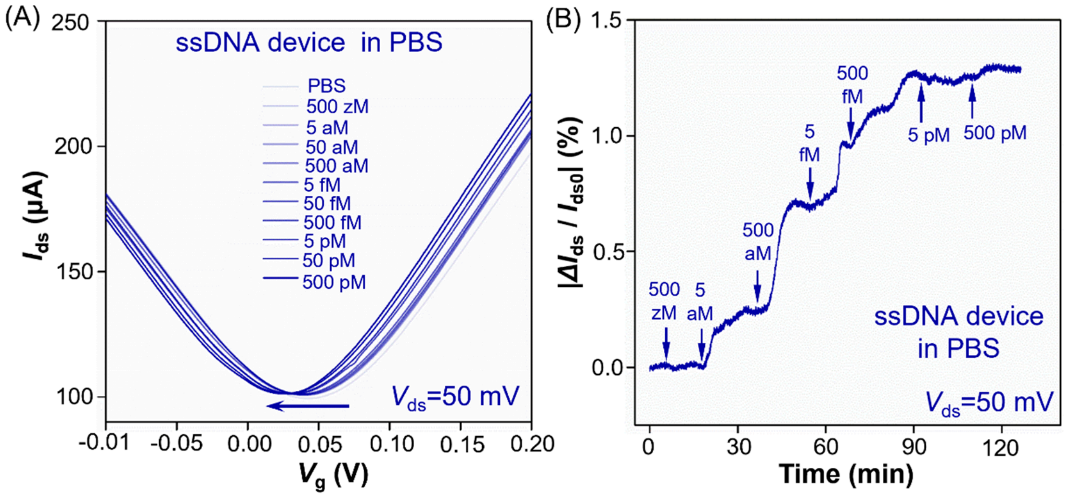

Figure S6: ssDNA probes modified G-FET device detection the insulin in PBS. (A), Transfer curve measurement of adding different concentration insulin in PBS (I_{ds}–V_g response curve) by the ssDNA probes modified G-FET device. **(B),** Real-time $|\Delta I_{ds}/I_{ds0}|$ response upon different insulin in PBS of the ssDNA probes modified G-FET device.

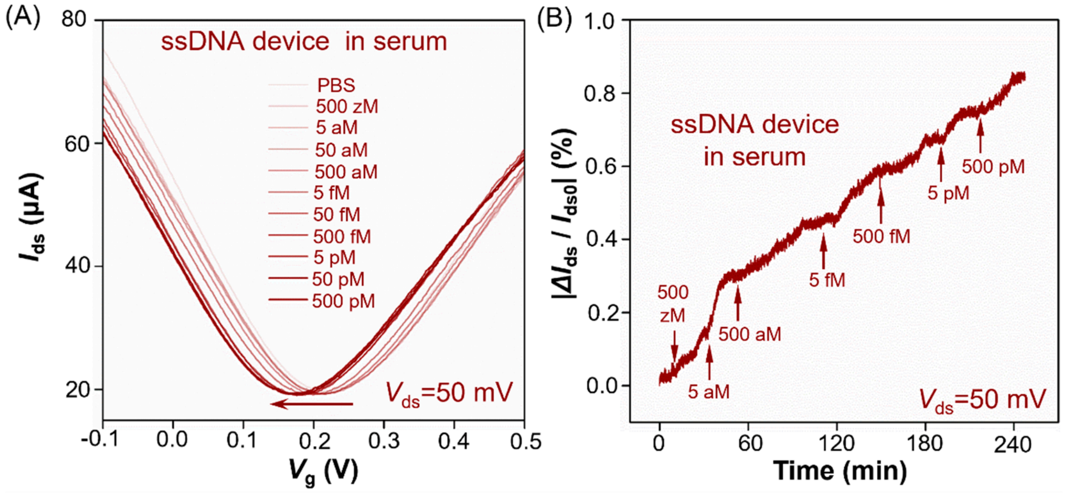

Figure S7: ssDNA probes modified G-FET device detection the insulin in serum. (A), Transfer curve measurement of adding different concentration insulin in serum (I_{ds}–V_g response curve) by the ssDNA probes modified G-FET device. **(B),** Real-time $|\Delta I_{ds}/I_{ds0}|$ response upon different insulin in serum of the ssDNA probes modified G-FET device.

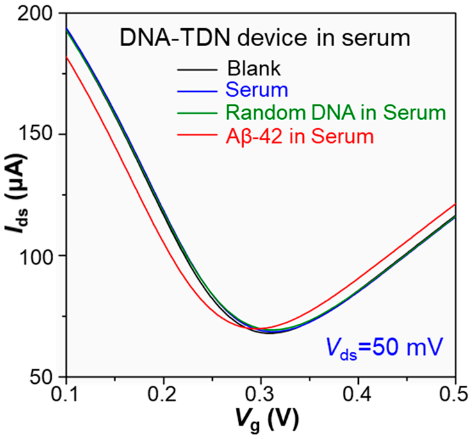

Figure S8: **Transfer curve measurement of adding serum, random DNA in serum and Aβ-42 in serum (I_{ds}−V_g response curve)**.

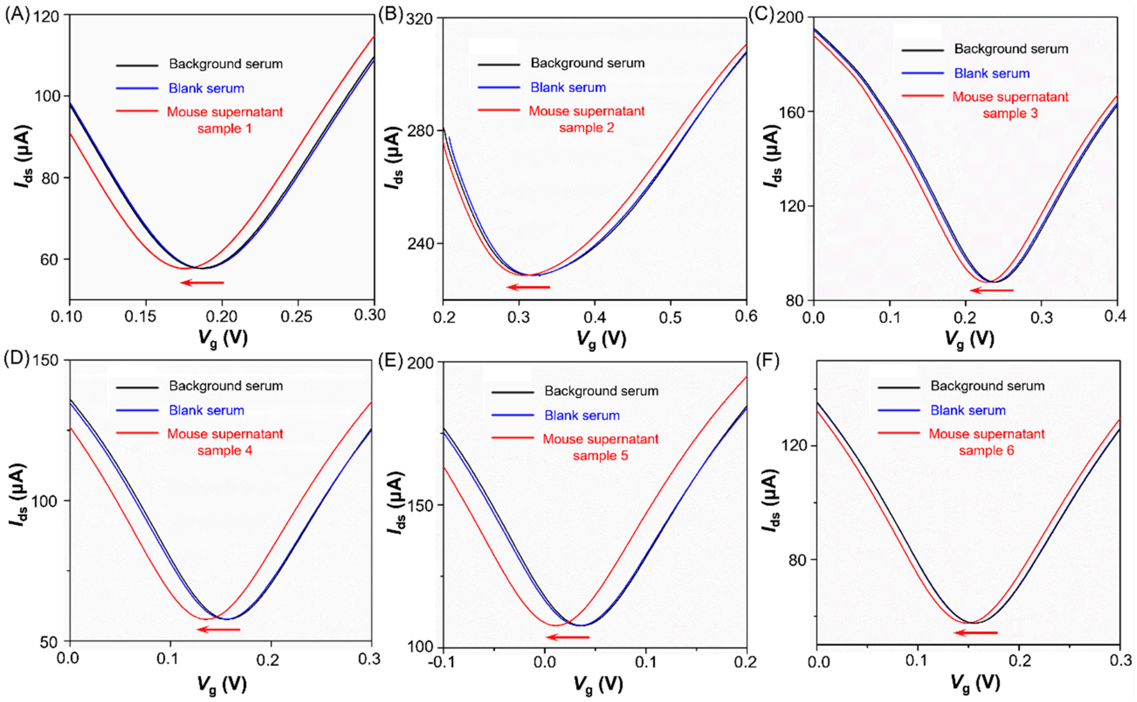

Figure S9: TDN G-FET device detection the insulin in supernatant samples from feeding mice. $|\Delta V_{\mathrm{Dirac}}|$ response of TDN G-FET sensor when adding different mouse supernatant samples (figure A, B,C, D, E and F are correspondenced Sample 1, 2, 3, 4, 5 and 6, respectively).

Table S1. The sequences (5′-3′) of DNA in this work.

ss-DNA	NH_2-C6-AGTCTAGGATTCGGCGTGGGTTAATTTTTTGCTGCCTGTGGTGTTGGGGCGGGTGCG-3′
NH_2-B17	NH_2-C6-TATCACCAGGCAGTTGACAGTGTAGCAAGCTGTAATAGATGCGAGGGTCCAATAC-3′
NH_2-C17	NH_2-C6- TCAACTGCCTGGTGATAAAACGACACTACGTGGGAATCTACTATGGCGGCTCTTC-3′
NH_2-D17	NH_2-C6-TTCAGACTTAGGAATGTGCTTCCCACGTAGTGTCGTTTGTATTGGACCCTCGCAT-3′
A17-5T	AGTCTAGGATTCGGCGTGGGTTAATTTTTTGCTGCCTGTGGTGTTGGGGCGGGTGCGTTTTTACATTCCTAAGTCTGAAACATTACAGCTTGCTACACGAGAAGAGCCGCCATAGTA-3′
A17-5T-Cy3	Cy3-AGTCTAGGATTCGGCGTGGGTTAATTTTTTGCTGCCTGTGGTGTTGGGGCGGGTGCGTTTTTACATTCCTAAGTCTGAAACATTACAGCTTGCTACACGAGAAGAGCCGCCATAGTA-3′

Table S2. ΔV_{Dirac} values measured by the TDN G-FET sensor for insulin test from the supernatant of experimental mice bloods.

**Samples**	ΔV_{Dirac} (mV)	ΔV_{Dirac} (mV)	ΔV_{Dirac} (mV)	**Samples**	ΔV_{Dirac} (mV)
S1	12	10	9	Blank serum 1	1
S2	14	16	19	Blank serum 2	3
S3	10	8	8	Blank serum 3	2
S4	20	18	24	Blank serum 4	3
S5	24	28	32	Blank serum 5	4
S6	18	12	20	Blank serum 6	3
				Blank serum 7	2
				Blank serum 8	4
